# Abstracts from the Student Medical Summit 2023

**DOI:** 10.1186/s12919-023-00278-7

**Published:** 2023-11-10

## A1. “It gave me a lot of hope”: The perspectives and experiences of mothers of pediatric heart transplant recipients who participated in a mindfulness-based retreat

### Mowa Ayibiowu^1,2^, Jia Lin^2^, Izabelle Siqueira^2^, Sarah J. Pol^2^, Taylor Robertson^3^, Joanna Mitchell^4^, Sara Ahola Kohut^2,5^, Ani Jamyang Donma^6^, Mirna Seifert-Hansen^7^, Heather Telfer^3,7^, Samantha J Anthony^2-4,7,8^

#### ^1^School of Medicine, University College Dublin, Dublin, Ireland; ^2^Child Health Evaluative Sciences, Peter Gilgan Centre for Research and Learning, The Hospital for Sick Children, Toronto, Ontario, Canada; ^3^Department of Social Work, The Hospital for Sick Children, Toronto, Ontario, Canada; ^4^Canadian Donation and Transplantation Research Program, Edmonton, Alberta, Canada; ^5^Department of Psychology, The Hospital for Sick Children, Toronto, Ontario, Canada; ^6^Spiritual and Religious Care Department, The Hospital for Sick Children, Toronto, Ontario, Canada; ^7^Transplant and Regenerative Medicine Centre, The Hospital for Sick Children, Toronto, Ontario, Canada; ^8^Factor-Inwentash Faculty of Social Work, University of Toronto, Toronto, Ontario, Canada

##### **Correspondence:** Mowa Ayibiowu (mowaayibiowu@gmail.com)

*BMC Proceedings 2023,* **17(Suppl 18):**A1

**Background**

Mothers are often the primary caregiver of pediatric heart transplant recipients. A mother’s ability to cope with the impact of their child’s dynamic health status influences familial, psychosocial and health outcomes. It is therefore critical that maternal coping is supported in pediatric healthcare. A mindfulness-based retreat (MBR) is proposed as an intervention to enhance community-building, mental health and quality of life in this population.

**Objective**

This study aims to explore the perspectives and experiences of mothers who attended a MBR.

**Methods**

A two-day MBR was piloted with mothers of pediatric heart transplant recipients from a Canadian pediatric heart transplant centre. The MBR consisted of mindfulness-based teachings, including meditation, deep relaxation and circle sharing. Qualitative data was collected through focus group interviews on the last day of the MBR and semi-structured individual interviews three months following the MBR. Interviews were audio-recorded, transcribed verbatim and subjected to thematic analysis.

**Results**

Sixteen mothers participated in the MBR held in April 2022 at a resort in Canada. Three preliminary themes emerged from analysis: (1) The importance of connecting and sharing stories with other mothers, (2) The value of learning mindfulness-based skills and building coping capacity and (3) The transformative nature of the MBR on mothers’ mental health and quality of life.

**Conclusion**

Findings support the MBR as an impactful intervention for mothers of pediatric heart transplant recipients that promotes family-centred care. Next steps include investigating the feasibility and clinical effectiveness of the MBR and the potential expansion to other clinical caregiving populations.

## A2. A systematic review comparing the different fixation approaches in the treatment of femoral neck fractures

### Hannah Beattie

#### Queen’s University Belfast

*BMC Proceedings 2023,* **17(Suppl 18):**A2

**Background**

Emergency departments are experiencing an ever-growing number of presentations of neck of femur (NOF) fractures [1]. In order to achieve stable fixation, there are three main internal fixation methods used when managing femoral neck fractures. These are cannulated screws (CS), sliding screws or dynamic hip screws (DHS) and proximal femoral locking plates (PFLP) [2]. The optimal treatment method has been continuously debated in the literature. The paper aims to critically review the current literature and compare the clinical outcomes of the most common methods, CS and DHS.

**Materials and methods**

A literature search of the databases PUBMED and SCOPUS was performed from inception to the present. Following the screening of the journal articles, nine articles were identified as eligible to be included in the review.

**Results**

The outcomes that emerged as the most commonly measured included infection, avascular necrosis (AVN), non-union and reoperation rates, the Harris hip score (HHS), the length of femoral neck shortening, surgery length and the amount of blood lost.

**Conclusions**

The DHS overall provided better functional outcomes for the hip joint in terms of the HHS. DHS also demonstrated more optimal results in relation to AVN, union, femoral neck shortening and reoperation rates. The CS implant had lower infection rates, shorter surgery times and a smaller amount of blood lost. However, there was inevitable heterogeneity between the studies in relation to how the outcomes were measured. The sample sizes are small and more prospective studies are required to gather stronger evidence to prove this result.

**References**

1. Chou C-T, Chou C-C, Law Y-Y, Lin Y-R. Hip fractures in patients admitted to emergency departments may increase the risk of acute affective disorders: A national population-based study. Journal of Acute Medicine. 2014;139–44.
2. Rajnish RK, Srivastava A, Rathod PM, Haq RU, Aggarwal S, Kumar P, et al. Does the femoral neck system provide better outcomes compared to cannulated screws fixation for the management of femoral neck fracture in young adults? A systematic review of literature and meta-analysis. Journal of Orthopaedics. 2022; 32:52–9.

## A3. The effects of reminder and information letters on non-attendance to a diabetic retinopathy screening clinic for pregnant patients

### Aditi Chaturvedi^2^, Stephen Kelly^2^, Karen O’Connor^1^, Ian Brennan^1^, Louise O’Toole^1^

#### ^1^Department of Ophthalmology, Mater University Hospital, Dublin, Ireland; ^2^School of Medicine, University College Dublin, Dublin, Ireland

*BMC Proceedings 2023,* **17(Suppl 18):**A3

**Background**

Sight-threatening diabetic retinopathy may be asymptomatic. Pregnancy is known to accelerate diabetic retinopathy. [1] Regular attendance to a diabetic retinopathy screening programme (DRS) during pregnancy is essential to detect and manage retinal pathology. [1] This audit aims to review whether sending a reminder and information letter to pregnant women due to attend DRS has any impact on nonattendance rates. The information letter described what to expect during the appointment and advised patients of what precautions to take prior to and post their screening.

**Materials and methods**

This was a retrospective comparative analysis of pregnant patients who missed at least one DRS appointment (non-attenders). The groups were divided into those who did not receive a reminder or information letter between April and August 2019, and those who received a letter one week prior to their appointment between April and October 2022. A subset of this patient cohort was defined as never-attenders. After gathering data on the characteristics of the patients, we performed statistical analyses on all groups to assess for differences.

**Results**

In 2019, 58 out of 127 patients (46%) did not attend their scheduled appointments. Following the introduction of a reminder and information letter in 2022, 15 out of 57 patients did not attend (26%). This finding achieved statistical significance (p=0.04). The mean age for both groups was 34 years, and the social class was marginally above average, assessed using the Pobal Deprivation Index. Non-attenders had a statistically significant lower median diabetes duration- those who had diabetes for longer were less likely to miss appointments (Figure 1). There was no significant difference in type of diabetes or distance to the screening centre. Reminder and information letters did not show any statistically significant impact on reducing the rate of never attendance.

**Conclusions**

The positive impact of both patient education and reminding our patients of their appointments is clearly demonstrated in this audit. Improved attendance rates benefit our patients’ ocular health and allow for better allocation of healthcare resources. We also identified a subset of patients who did not attend DRS, further analysis of this group is warranted to identify potential barriers to patient engagement with the diabetic retinopathy screening programme.

**References**

1. Morrison, J.L. *et al.* (2016) “Diabetic retinopathy in pregnancy: A Review,” *Clinical & Experimental Ophthalmology*, 44(4): 321–334. Available at: https://doi.org/10.1111/ceo.12760.

**Fig. 1 (abstract A3).**
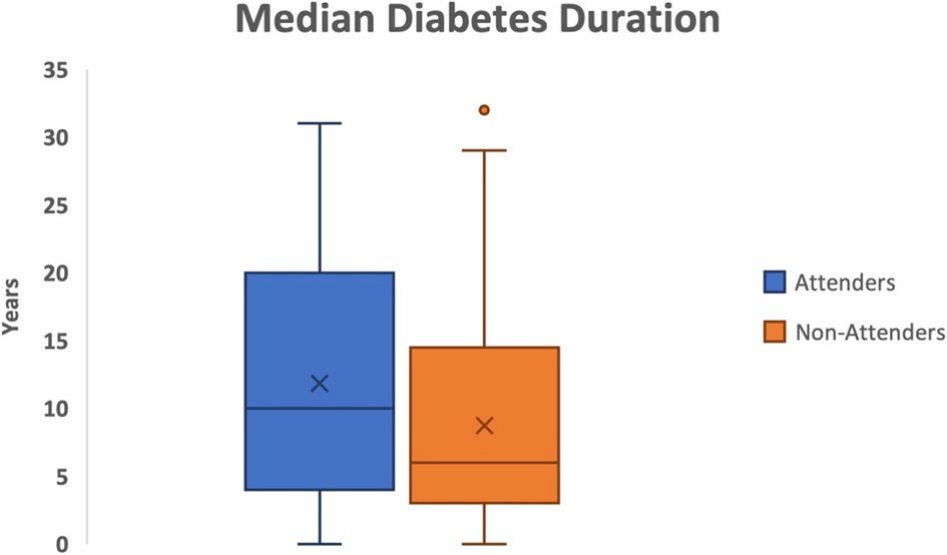
Median Duration of Diabetes (IQR): Attenders- 10 (4,20), Non Attenders- 6 (3, 13.5). P = 0.02

## A4. A systematic review of the efficacy of ultrasound guided fascia iliaca nerve block versus landmark guided fascia iliaca block with regards to analgesic control

### Orla Coleman^1^, Alan Watts^2^

#### ^1^University of Limerick, Ireland; ^2^Emergency Department, University Hospital Limerick, Ireland

**Correspondence:** Orla Coleman (orlamcoleman@gmail.com)

*BMC Proceedings 2023,* **17(Suppl 18):**A4

**Background**

Hip fractures are commonly seen in the emergency department [1]. Regional anaesthesia can form part of the analgesic plan and fascia iliaca compartment block (FICB) is a recommended choice [2]. The aim of this study was to conduct a systematic review of the efficacy of ultrasound-guided (US) FICB versus landmark-guided FICB with regards to analgesic control in patients with a neck of femur fracture. A scientifically supported consensus on how best to conduct the FICB has not been established.

**Methods**

A systematic review adhering to PRISMA guidelines was undertaken, searching five electronic databases Medline (PUBMED, OVID and EBSCO), Scopus and Cochrane Library. Search headings included ‘hip fracture’, ‘femoral neck fracture’, ‘analgesia’, ‘pain’ and ‘nerve block’. English full-text randomised control trials that reported a pain outcome following a FICB were included. Studies were excluded based on study design, paediatric population, comparison of FICB with another nerve block, or if it did not meet the primary endpoint or were animal or cadaveric studies. Suitable publications were assessed for risk of bias using the Rob2 Tool for randomised control trials. The primary outcome was a documented pain score following FICB.

**Results**

This study identified 1095 studies, and after examination, 10 were deemed suitable for inclusion in the review. A total of 840 patients were identified across the studies. No study directly compared US-guided FICB with landmark-guided FICB. 3/4 studies that used the US-guided technique had statistically significant pain reductions scores following the FICB. 2/6 studies that used the landmark-guided technique had statistically significant pain reduction scores following the FICB. Four studies showed that opioid consumption was less following a FICB.

**Conclusion**

There was not sufficient data to conclude that one technique is superior to the other. Further high-quality research is required to draw this conclusion.

**References**

1. Ahern, E., Brent, L., Connolly, A., Ferris, H., Hurson, C., Kelly, F. and Savin, B. (2021) *Irish Hip Fracture Database National Report 2020*: National Office of Clinical Audit.
2. Murphy, R. (2018) *Fascia Iliaca Compartment Block for Proximal Femur Fracture in the Emergency Department* Irish Association for Emergency Medicine.

## A5. Parental perceptions of neonatal clinical research: a systematic review

### Yasir Alshareefy^1 †^, Muhammad Amirsalman Bin Nor Azman^1†^, William Choong^1†^, Emily Doyle^1†^, Shamik Giri^1†*^, Garrett Huwyler^1†^, Guillaume Lambert^1†^, Bianca Mascan^1†^, Harry Sheehy^1†^, Oyindunmola Sodeke^1†^, David Mockler^2^, Judith Meehan^1,3,4^, Eman Isweisi^1^, Philip Stewart^1^, Aoife Branagan^1,8^, Edna Roche^1,4,5^, Eleanor J Molloy^1,3-8^

#### ^1^Discipline of Paediatrics, Trinity College Dublin, the University of Dublin, Dublin, Ireland; ^2^ John Stearne Medical Library, Trinity College Dublin, St James Hospital, Dublin, Ireland; ^3^Trinity Translational Medicine Institute (TTMI), St James Hospital & ^4^Trinity Research in Childhood Centre (TRiCC), Dublin, Ireland; ^5^Endocrinology, Children’s Health Ireland (CHI) at Tallaght, Dublin, Ireland; ^6^ Neurodisability, Children’s Health Ireland (CHI) at Tallaght, Dublin, Ireland; ^7^Neonatology, CHI at Crumlin, Dublin, Ireland; ^8^Paediatrics, Coombe Women’s and Infants University Hospital, Dublin, Ireland

##### **Correspondence:** Shamik Giri (giris@tcd.ie)

*BMC Proceedings 2023,* **17(Suppl 18):**A5

^†^Authors contributed equally and are co-first authors

**Background**

Clinical research is integral to developing new therapies for the vulnerable neonatal population. There is an extensive range of factors influencing parental perceptions of neonatal clinical research. An appraisal of these factors may aid researchers to better recruit participants. This systematic review aimed to explore parental attitudes and opinions of conducting clinical research in their infants.

**Materials and Methods**

A search strategy was generated in collaboration with an experienced medical librarian. This strategy was subsequently applied to the Embase, MedLine, Web of Science, and CINAHL databases. Data was extracted and screened according to PRISMA guidelines.

**Results**

The search yielded 21 papers from over 1250 parents across multiple geographic regions. Three primary themes of parental perceptions towards clinical research were identified, namely outcomes of research, parental expectations, and barriers/difficulties, as illustrated in Figure 1. The key motivators of parental participation included perceived personal gain, contribution to scientific knowledge, altruism, and increased family support from the healthcare team. We also identified some major barriers to research involvement, such as overwhelming participation burden, high levels of emotional distress, poorly timed enrolment discussions, and poor comprehension and retention of pertinent information.

**Conclusion**

A variety of factors exist that influence parental perceptions of paediatric research, ranging from personal concerns and clinician influences to circumstantial difficulties and societal impacts. The continued acknowledgement of these parental concerns, and the empowerment of families to make informed decisions, can only serve to improve clinical research outcomes. **Figure 1**. Thematic grouping of parental perceptions towards neonatal research

**Acknowledgements**

The first authors of this paper are medical students from Trinity College Dublin, The University of Dublin, Ireland.

**Fig. 1 (abstract A5).**
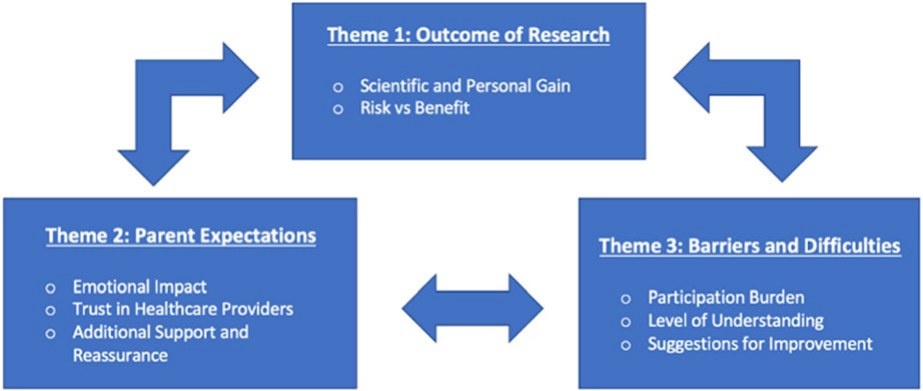
Thematic grouping of parental perceptions towards neonatal research

## A6. Pulmonary artery pulsatility index is inversely associated with mortality in patients with significant tricuspid valve regurgitation

### Conor J. Kane^1,2^, Kyla M. Lara-Breitinger^2^, Vuyisile T. Nkomo^2^, Ratnasari Padang^2^, Cristina Pislaru^2^, Grace Lin^2^, Sorin V. Pislaru^2^

#### ^1^ School of Medicine, University College Dublin, Dublin, Ireland; ^2^ Department of Cardiovascular Diseases, Mayo Clinic, Rochester, MN, USA

*BMC Proceedings 2023,* **17(Suppl 18):**A6

**Background**

Tricuspid valve regurgitation (TR) is a common valvular disease associated with increased mortality. Pulmonary artery pulsatility index (PAPi) has emerged as a hemodynamic risk predictor in left heart disease and in pulmonary hypertension (PH). Whether PAPi discriminates risk in unselected patients with significant TR is unknown.

**Materials and Methods**

This was a retrospective consecutive cohort of patients with ≥ moderate TR (regardless of aetiology) on their first echocardiogram with PA pressure measurements. Exclusion criteria were previous tricuspid valve intervention and declined research authorization. PAPi was calculated as (PA systolic pressure - PA diastolic pressure)/right atrial pressure. The primary outcome was all-cause mortality at 5 years. Patients were categorized by presence/absence of PH (PA systolic pressure ≥ 50 mm Hg) and by PAPi levels.

**Results**

Of the 5,079 patients studied, 2,741 (54%) had PH. The median PAPi was 3.0 (IQR 1.9, 4.4). Both the presence of PH and decreasing levels of PAPi were associated with larger right ventricles, worse right ventricular systolic function, higher NT-pro BNP levels, greater degrees of heart failure and worse survival (Figure 1).

**Conclusions**

In patients with significant TR with and without PH, lower PAPi is associated with right ventricular dysfunction, heart failure, and worse survival. Incorporating PA pressure and PAPi may help stratify disease severity in patients with significant TR regardless of aetiology.

**Fig. 1 (abstract A6).**
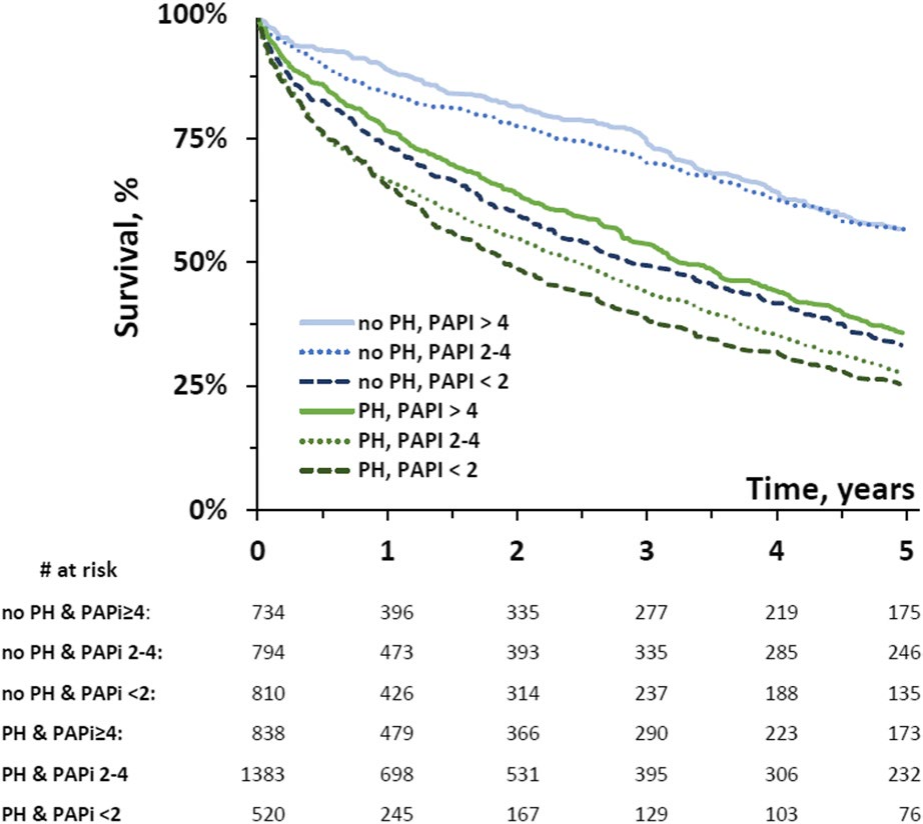
Kaplan-Meier survival curve demonstrates survival from all-cause mortality over 5 years in those patients with significant TR subdivided based on the presence or absence of PH and their PAPi levels

## A7. Analysing the role of SERPINE1 network in the pathogenesis of human glioblastoma

### Zahra Khosravi^1, 2*,^ Chandrasekaran Kaliaperumal^3^ and Arun HS Kumar^2^

#### ^1^School of Medicine & Medical Science, University College Dublin, Belfield, Dublin-04, Ireland; ^2^Stemcology, School of Veterinary Medicine, University College Dublin, Belfield, Dublin-04, Ireland; ^3^Department of Clinical Neurosciences, Royal Infirmary of Edinburgh, Edinburgh, United Kingdom

##### **Correspondence:** Zahra Khosravi (zahra.khosravi@ucdconnect.ie)

*BMC Proceedings 2023,* **17(Suppl 18):**A7

**Background**

Glioblastoma multiforme (GBM) is an aggressive brain tumour with a 5-year survival rate of less than 6%^[1,2]^. SERPINE1 is a novel GBM receptor that modulates its progression through growth signals and extracellular matrix remodelling ^[1,3]^. We recently reported phytochemicals from *Calotropis gigantea* as potential anticancer therapeutic leads ^[4]^. We investigated the role of SERPINE1 network proteins in GBM pathogenesis, and assessed targetability with the selected *C.Gigantea* phytochemicals.

**Material and methods**

SERPINE1 network proteins were identified using String Database [https://string-db.org], and affinities analysed using Chimera software. SERPINE1 expression in brain parenchyma was evaluated to correlate its relevance to GBM using the Human Protein Atlas Database [https://www.proteinatlas.org]. Select phytochemicals from *C.gigantea* were screened using AutoDock Vina to assess SERPINE1 targetability.

**Results**

VTN, PLG, TGFB1, VWF, FGF2 and CXCR1 were identified as major SERPINE1 network proteins. The strongest interaction was observed between SERPINE1 and FGF2, and CXCR1. Our results suggest they play a role in GBM progression through brain parenchyma by creating a prime carcinogenesis microenvironment. The highest SERPINE1 expression was in the brainstem, corpus callosum and spinal cord. This expression was consistent with high-grade GBM. The selected *C.Gigantea* phytochemicals were observed to have therapeutic binding affinity and predicted efficacy against SERPINE1(table 1).

**Conclusion**

SERPINE1 plays a vital role in GBM progression through its network proteins. Currently, temozolomide is a first-line treatment option, however most responding GBM recur ^[2,3]^. Phytochemicals from *C.gigantea* tested can serve as lead compounds for developing novel anti-SERPINE1 therapeutics for GBM.

**Acknowledgements**

Research support from University College Dublin-Seed funding/Output Based Research Support Scheme (R19862, 2019), Royal Society-UK (IES\R2\181067, 2018) and Stemcology (STGY2917, 2022) is acknowledged.

**References**

1. Seker, F., Cingoz, A., and Bagci-Onder, T., et al. Identification of SERPINE1 as a regulator of glioblastoma cell dispersal with transcriptome profiling. Cancers, , 2019, 11(11):1651-54.
2. Woroniecka, K., Chongsathidkiet, P., and Rhodin, K., et al. T-Cell Exhaustion Signatures Vary with Tumor Type and Are Severe in GlioblastomaT-Cell Exhaustion Signatures in Glioblastoma. . Clin Cancer Res. 2018, 4175-86.
3. Shergalis, A., Bankhead, A., and Luesakul, U., et al. Current Challenges and Opportunities in Treating Glioblastoma. Pharmacol Rev, 2018, 70:412-45.
4. Khosravi Z and Kumar, A.H. Pharmacognosy and pharmacology of Calotropis gigantea for discovery of anticancer therapeutics. Pharmacog Mag., 2021, Apr 1;17(6):123-27.

**Table 1 (abstract A7).** Binding affinity of *C.Gigantea* phytochemicals and Imatinib with SERPINE1

	Binding affinity with SERPINE1	
	K_**i**_(μM)^**a**^	IC_**50**_(μM)^**b**^
Nicotiflorin	329.6 ± 3.6	659.2 ± 6.1
Mefruside	557.8 ± 2.6	1115.7 ± 8.7
Quercetin	146.1 ± 5.6	292.8 ± 11.3
Zingerone	477.7 ± 4.2	955.1 ± 6.4
Imatinib^c^	858.8 ± 6.5	1717.7 ± 9.1

^a^K_i_ is the binding affinity of *C.Gigantea* phytochemicals and imatinib with SERPINE1

^b^IC_50_ is the efficacy of the *C.Gigantea* phytochemicals and imatinib to inhibit SERPINE1

^c^Imatinib was used as a reference compound

## A8. Rare dermatological toxicities of pembrolizumab: a case series

### Miriam Matthews^1^, Josh Matthews^2^, Mathews George^3^

#### ^1^University College Dublin, Dublin, Ireland; ^2^University of Central Lancashire, Preston, Lancashire, UK; ^3^Nacogdoches Cancer Care Associates, Nacogdoches, Texas, USA

##### **Correspondence:** Miriam Matthews (miriam.matthews@ucdconnect.ie)

*BMC Proceedings 2023,* **17(Suppl 18):**A8

**Background**

Pembrolizumab is a PD-L1 inhibitor used in the treatment of malignant melanoma, lung cancers, and other malignancies. Immunotherapies such as Pembrolizumab have been associated with rare, severe dermatological side effects such as vitiligo, psoriasis, dermatomyositis, Stevens-Johnson Syndrome (SJS) and bullous pemphigoid. This case series demonstrates two patients developing vitiligo and bullous pemphigoid as an immune related adverse effect of Pembrolizumab treatment.

**Case report**

A 63-year-old man with metastatic stage IV melanoma underwent treatment for five months with pembrolizumab. Following his 5th cycle patient presented with discoloration of his hands and scalp consistent with vitiligo (Figure 1). The patient however was tolerating immunotherapy well and had no prior history of vitiligo or other autoimmune conditions. A Follow-up PET scan at six months showed no hypermetabolic activity, indicating no active disease. The appearance of vitiligo remained unchanged at six months follow-up.

A 74-year-old man with renal clear cell carcinoma was treated with adjuvant pembrolizumab post nephrectomy. 5 months following initiation with Pembrolizumab, the patient developed oedematous, papulo-vesicular, and bullous eruptions widely distributed on the legs, hands, feet, abdomen, and back (Figure 2). The lesions began as an itchy sensation on the back that spread to the extremities, which progressed to blisters. A punch biopsy by dermatology revealed bullous pemphigoid. Pembrolizumab was discontinued and the patient began treatment with 60mg of prednisone. Two months following steroid treatment, the lesions had resolved and the patient was left with a mild itching and burning sensation of the hands and discoloration of the palms and wrists.

Bullous pemphigoid and vitiligo are well-established immune-related adverse events, due to the unique mechanism of action of checkpoint inhibitor therapy. While the etiologies are unclear, both toxicities are related to a breakdown in immune tolerance: immunotherapy-induced vitiligo has been suggested to be related to release of melanocytic antigens from immune-therapy-mediated tumor destruction; bullous pemphigoid could be related to the inhibition of immunosuppressive B cells.^Ellis^

The incidence of immunotherapy induced vitiligo may be associated with improved treatment response and survival. A history of use of immune checkpoint inhibitor therapy being essential to confirm that the toxicities are indeed immunotherapy-induced.

**Conclusion**

While bullous pemphigoid and vitiligo-like lesions following immunotherapy are rare, it is important for clinicians to recognize them, as they potentially can be associated with a positive response to treatment. Further studies should explore this association.

**Acknowledgments**

Both patients provided informed consent to the publication of their information and images in Figures 1 and 2.

**References**

1. Ellis SR, Vierra AT, Millsop JW, Lacouture ME, Kiuru M. Dermatologic toxicities to immune checkpoint inhibitor therapy: A review of histopathologic features. Journal of the American Academy of Dermatology [Internet]. 2020 Oct [cited 2022 Dec 31];83(4):1130–43. Available from: https://www.ncbi.nlm.nih.gov/pmc/articles/PMC7492441/
2. Postow MA, Sidlow R, Hellmann MD. Immune-Related Adverse Events Associated with Immune Checkpoint Blockade. Longo DL, editor. New England Journal of Medicine. 2018 Jan 11;378(2):158–68.
3. Lopez AT, Khanna T, Antonov N, Audrey-Bayan C, Geskin L. A review of bullous pemphigoid associated with PD-1 and PD-L1 inhibitors. International Journal of Dermatology. 2018 Apr 6;57(6):664–9.

**Fig. 1 (abstract A8).**
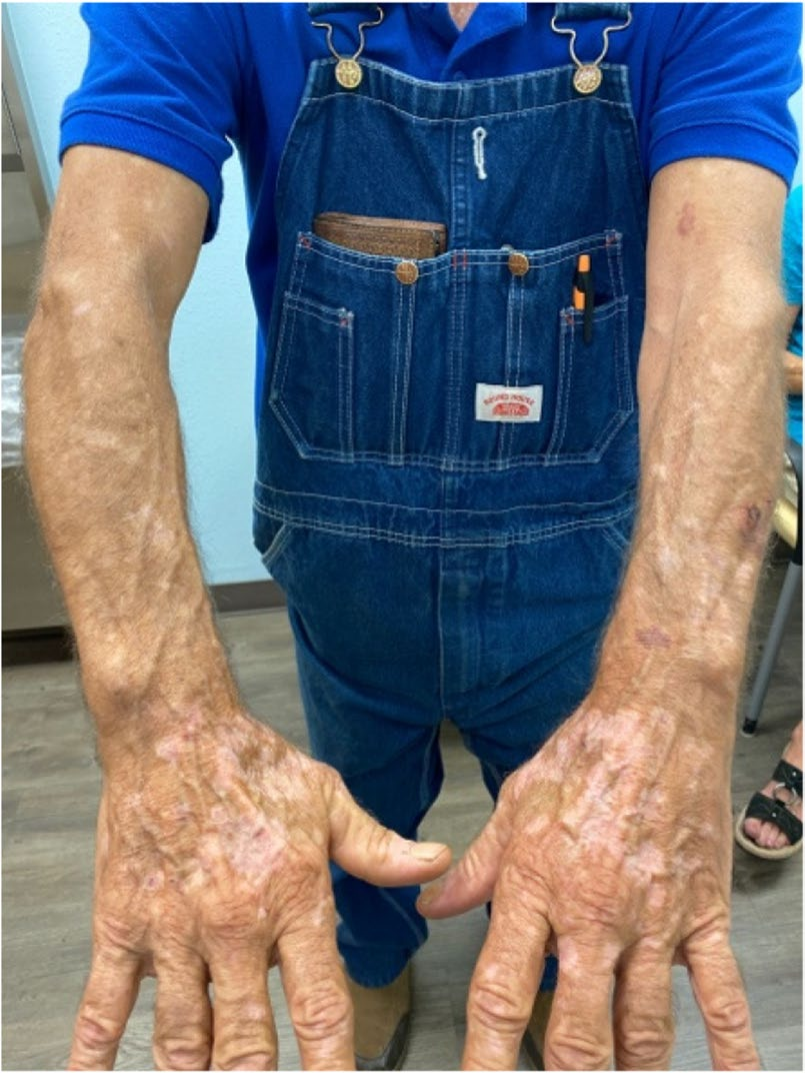
Immunotherapy-induced vitiligo

**Fig. 2 (abstract A8).**
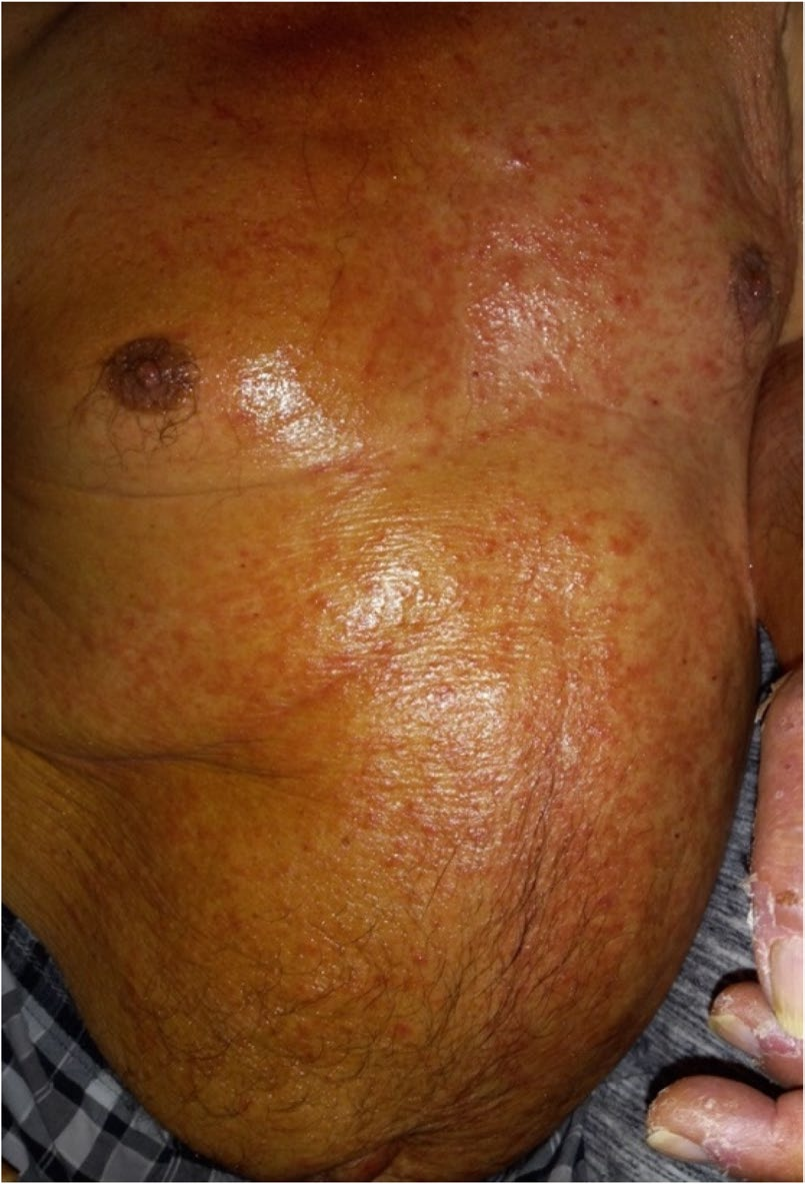
Bullous pemphigoid

## A9. Gender bias in North American obstetrics and gynecology conferences

### Ainsley Matthewson^1^, Ryan Chow^2,3,4^, Georges Khalaf ^5^, Nandini Biyani ^6^, Sejal Khandelwal^7^, Evan Tannenbaum^3,8^

#### ^1^ School of Medicine, University College Dublin, Belfield, Dublin; ^2^ Faculty of Medicine, University of Ottawa, Ottawa, Ontario; ^3^ Lunenfeld-Tanenbaum Research Institute, Department of Obstetrics and Gynecology, Mount Sinai Hospitals, Toronto; ^4^Clinical Epidemiology Program, Ottawa Hospital Research Institute, Department of Obstetrics and Gynecology, University of Ottawa, Ottawa; ^5^ Faculty of Engineering, University of Ottawa, Ottawa, Ontario; ^6^ Faculty of Science, University of Ottawa, Ottawa, Ontario; ^7^ Faculty of Science, University of Western, London, Ontario; ^8^ Faculty of Medicine, University of Toronto, Toronto, Ontario

##### **Correspondence:** Ainsley Matthewson (ainsley.matthewson@ucdconnect.ie)

*BMC Proceedings 2023,* **17(Suppl 18):**A9

**Background**

Female representation in obstetrics and gynecology (ob/gyn) meetings is important for gender equity. Our objective was to examine if a gender bias exists and to determine whether male or female faculty of equal academic merit were offered equal opportunities to speak at major North American ob/gyn conferences.

**Materials and methods**

Conferences and presenter information were electronically retrieved. Conference information extracted involved: name, organizing institution, number of sections, and duration. Presenter information was extracted in duplicate and independently. Presenter information extracted involved: name, speaking time, h-index, number of publications, number of citations and years of practice. Gender was determined with online software Genderize.io. Conflicts were resolved with a third reviewer. One-way ANOVA was conducted to evaluate the difference in parametric variables and the Mann-Whitney U test was utilized to evaluate the differences in medians.

**Results**

Male and female had significant differences regarding talking time when academic merit was taken into account. The mean talking time for female vs. male was 29.9 and 30.8 (SEM: 1.2 ± 1.7, 95% CI: -2.2 to 4.7). Mean years of practice for female vs. male was 16.7 and 25.3 (SEM: 8.6 ± 1.1, 95% CI: 6.4 to 10.9). Mean number of publications for female vs. male was 40.5 and 84.0 (p< 0.01). Median number of citations for female vs. male was 707.5 and 1582 (p<0.01). Median h-index for female vs. male was 12.0 and 19.50 (p<0.01).

**Conclusions**

Our study showed that there is a gender bias present in North American ob/gyn conferences, where being a male is associated with less talking time than females of equal academic merit. Further studies are needed to elucidate the true effects of gender on opportunities at ob/gyn meetings.

## A10. Differences in muscle mass regulation depending on the timing of *Bmal1* deletion

### Charlotte McCreery^1,2^**,** Ronan Lordan^2,3,4^, Matthew Thomas^1,2^, Sarah L. Teegarden^2,3,4^, Soon-Yew Tang^2,3,4^, Carsten Skarke^2,3,4^ and Garret A. FitzGerald^2,3,4^

#### ^1^UCD School of Medicine, University College Dublin, Belfield, Dublin, Ireland; ^2^Institute for Translational Medicine and Therapeutics, Perelman School of Medicine, Philadelphia, PA, USA; ^3^Department of Systems Pharmacology and Translational Therapeutics, Perelman School of Medicine, University of Pennsylvania, Philadelphia, PA, USA; ^4^Department of Medicine, Perelman School of Medicine, University of Pennsylvania, Philadelphia, PA, USA

##### **Correspondence:** Garret A. FitzGerald (garret@upenn.edu); Ronan Lordan (ronan.lordan@pennmedicine.upenn.edu)

*BMC Proceedings 2023,* **17(Suppl 18):**A10

**Abstract**

*Bmal1* is an essential core clock gene that governs daily rhythmic gene expression. Investigating the dysregulation of the skeletal muscle circadian clock is important to determine its role in the onset of metabolic and skeletal muscle diseases including obesity, type II diabetes, and sarcopenia. We assessed the timing of *Bmal1* deletion utilising embryonic deletion (eKO) or global inducible postnatal deletion (iKO), on muscle phenotypes. Similar to previous studies, eKO *Bmal1* mice exhibited an accelerated aging phenotype [1] in muscle not induced in the iKO mice. To investigate these differential phenotypes, we assessed the molecular clock's role in muscle mass regulation. Histological examination displayed small muscle fibres (500-750 μm^2^) were present in greater quantities in the eKO mice and had reduced grip strength versus control mice, which was not observed in iKO muscle. A critical regulator of muscle atrophy, *Trim63*, was elevated in exercised iKO mice and controls as determined by RT-qPCR. However, *Trim63* was elevated to a greater extent in exercise-trained eKO mice versus exercised controls, suggesting higher levels of atrophy in the eKO mice versus the iKO mice. Additional regulators of atrophy, *Fbxo32* and *Fbxo21*, were also differentially expressed post-exercise in the eKO mice versus controls but not in the iKO mice. Collectively, these preliminary findings indicate that the eKO mice exhibit an atrophic muscle phenotype in part mediated by Trim63. Further research is required to uncover the role of *Bmal1* in skeletal muscle mass regulation in development, as we age, and in response to dysregulation of the clock.

**Acknowledgments**

The authors would like to acknowledge the support of the University College Dublin Dean’s Scholarship.

**References**

1.Schiaffino S, Blaauw B, Dyar K. A. The functional significance of the skeletal muscle clock: lessons from Bmal1 knockout models. Skeletal muscle*.* 2016; 6:1-9.

## A11. Bridging the Gap - Hand Hygiene Perception and Practice Discrepancies in Tanzania

### Rachel Picard^1^, Michael Mwandri^2^

#### ^1^School of Medicine, University College Dublin, Belfield, Dublin, Ireland; ^2^Health for a Prosperous Nation, Dar es Salaam, Tanzania

*BMC Proceedings 2023,* **17(Suppl 18):**A11

**Background**

As medicine advances exponentially, one concept remains fundamental - hand hygiene. Hand hygiene drastically improves patient outcomes and decreases rates of hospital-acquired infections, often catastrophic in low- and middle-income countries (LMICs) such as Tanzania.^1^ This study attempts to explain why hand hygiene practices remain poor, despite LMIC healthcare providers understanding its importance. This study was completed at Arumeru District Hospital in Tanzania.^2^

**Materials and Methods**

Hand hygiene knowledge, perception and practice were measured at Arumeru District Hospital using the WHO’s knowledge, perception, and observation surveys. The observation survey allowed for contemporaneous recording of hand hygiene practice. Ward infrastructure surveys were also utilised, along with a focus group, to observe infrastructure, measure knowledge and perceptions, and explore barriers.

**Results**

Basic hand hygiene was utilised in 9.2% of observed clinician-patient interactions. There was a heavy reliance on glove use which occurred in 57.3% of interactions compared to hand rub and handwashing being used during 6.9% and 7.6% respectively (Table 1). The observed results were incongruous with the Perception Survey, where respondents believed clinicians performed hand hygiene in 76.8% of required situations.

**Conclusion**

The results indicate disparities between expectation and action - clinicians articulated hand hygiene importance and believed it was practised, but observations refute these beliefs. Hand hygiene deficiency was explained by the infrastructure survey and qualitative interviews, which indicate severe deficiencies of resources. The findings indicate the need for sustained investments in infrastructure, as further hand hygiene training will have limited effect on improving practice and ultimately patient outcomes without the necessary resources.

**Acknowledgements**

This research is presented on behalf of Health for a Prosperous Nation. We sincerely and deeply thank the clinicians and administrators at Arumeru District Hospital for their warm welcome and participation in this research.

**References**

1. Habboush Y, Benham MD, Louie T, Noor A, Sprague RM. New York State Infection Control. In: StatPearls [Internet]. Treasure Island (FL): StatPearls Publishing; 2022. Available from: http://www.ncbi.nlm.nih.gov/books/NBK565864/
2. Allegranzi B, Gayet-Ageron A, Damani N, Bengaly L, McLaws ML, Moro ML, et al. Global implementation of WHO’s multimodal strategy for improvement of hand hygiene: a quasi-experimental study. The Lancet Infectious Diseases. 2013 Oct;13(10):843–51.

**Table 1 (abstract A11).** Questionnaire and observation results by respondent occupation

	All Participants	Medical Doctors and Clinical Officers	Other Health Care Workers	Significance (p)
Correct Hand Hygiene Procedure Used	9.2 %	11.1 %	8.4 %	-
Hand Rub Used	6.9 %	13.9 %	4.2 %	-
Handwashing Used	7.6 %	11.1 %	6.3 %	-
Gloves Used	57.3 %	52.8 %	59.0 %	-
Total Knowledge Score	56.3 %	56.0 %	56.5 %	-
Did you receive formal training on hand hygiene in the last three years?	66.7 %	100.0 %	50.0 %	.027 **
On average, in what percentage of situations requiring hand hygiene do healthcare workers in your hospital actually perform hand hygiene, either by handrubbing or handwashing?	76.8 %	66.0 %	82.1 %	-
What is the effectiveness of hand hygiene in preventing healthcare-associated infection? (1-4)	3.7	3.5	3.8	-

***, **, * denotes statistical significance at the 1, 5, and 10 percent levels respectively. No statistical significance is denoted by -

## A12. Utilisation of Dissections vs Prosections in Anatomical Education and its Relevance in Pursuing Surgical Training: A Comparative Study on Students’ Perspectives at a Northern Irish Medical School

### Sakshi Roy, Arjun Ahluwalia, Muhammad Hamza Shah

#### Queen’s University Belfast, Belfast, County Antrim, UK

##### **Correspondence:** Sakshi Roy (sroy06@qub.ac.uk)

*BMC Proceedings 2023,* **17(Suppl 18):**A12

**Aim**

This study investigates medical students’ opinions regarding their memorisation, retention and implementation of anatomical learning by comparing prosection and dissection studies offered at the university. Furthermore, we also investigated its significance in students considering the surgical training pathway in the future.

**Methods**

Medical students at QUB were sent a 10-item survey weighing their preference for dissection and prosection in the retention and application of anatomical knowledge along with its relevance in pursuing surgery. The survey was distributed amongst Year 1 - 5 medical students in an online format, with answers recorded over the span of two weeks.

**Results**

26 QUB medical students across preclinical/clinical years answered the survey, 53.85% of whom preferred dissections for identification, memorisation and employment of information regarding anatomical structures. Students who chose dissection agreed that the process offered more interaction, with deeper knowledge about spatial organ recognition. Meanwhile, prosections were chosen by students who believed focusing on isolated organs helped in mapping their knowledge to relevant learning outcomes.

Moreover, 42.11% of respondents found both learning methods helped visualise anatomical concepts outside a medical environment. However, dissection helped most students draw links between pathology and its anatomical basis. Finally, 84.21% considered dissection to play a major role in viewing surgery as a future career.

**Conclusion**

While there is an observable difference in respondents’ perspectives, the survey indicates an overall preference for dissection in anatomical lab practices. The practice appealed to students better due to its greater interactive experience and tactile approach, with respondents agreeing that dissection mimics true surgical craftsmanship.

## A13. Evaluating the perceptions and educational experiences of medical students during the Covid-19 pandemic

### Ernest Z Low, Niall J O’Sullivan, Vidushi Sharma, Isabella Sebastian, Roisin Meagher, Dalal Alomairi, Ebraheem H Alhouti, Claire L. Donohoe, Michael E Kelly

#### Trinity College Dublin, St. James’s Hospital, Dublin, Ireland

##### **Correspondence:** Vidushi Sharma (sharmavi@tcd.ie)

*BMC Proceedings 2023,* **17(Suppl 18):**A13

**Introduction**

The COVID-19 pandemic has had a significant impact on how traditional medical education is taught. Medical education programs have had to adapt quickly to digital learning and deal with limitations on face-to-face instruction. We want to see how this pandemic has affected the perceptions and experiences of medical students.[1]

**Methods**

Between March and April 2022, a cross-sectional survey of medical students at Trinity College Dublin (TCD) was carried out. The review investigated understudy fulfillment with the ongoing training program, showing conveyance and the impact

of Coronavirus on schooling and understudy prosperity.[1]

**Results**

The survey saw participation from 175 medical students. The majority of students were satisfied or neutral with their medical education as a whole. Tutorials and problem-based learning (PBL) were deemed the most effective teaching method by 93 participants (53.1%), followed by laboratory and clinical placements by 78 participants (44.6%), and hybrid learning by 85 participants (48.6%). The pandemic's effects on how education was delivered were met with mixed reactions. 67 participants (40.6 percent) were satisfied with the changes, 64 participants (38.8 percent) were neutral, and only 34 participants (20.6%) were dissatisfied. However, with 96 participants (55.8%) reporting negative responses, the majority of participants believed the pandemic had a negative impact on their mental health. [1]

**Conclusion**

While digital content and delivery offer the advantage of greater flexibility in learning, they do not offer the advantage of classroom instruction or hands-on training. The potential advantages of online education are bolstered by our findings.[1]

**References**

1. Low EZ, O'Sullivan NJ, Sharma V, Sebastian I, Meagher R, Alomairi D, Alhouti EH, Donohoe CL, Kelly ME. Assessing medical students’ perception and educational experience during COVID-19 pandemic. Ir J Med Sci. 2022.

## A14. Suite 4 Health information technology systems evaluation following implementation for usability, effectiveness and patient safety

### Kristen L Snyder^1^, Dr. Roisin Tully^2^

#### ^1^School of Medicine, University College Dublin, Belfield, Dublin, Ireland; ^2^Department of Breast, Endocrine, and General Surgery of St. Vincent’s University Hospital, University College Dublin, Dublin, Ireland

*BMC Proceedings 2023,* **17(Suppl 18):**A14

**Background**

Implemented health information technology (HIT) infrastructure must meet information needs of clinical hospital staff for the safe and effective management of patients [1,2], and teams maintaining HIT systems require an understanding of the specific needs of clinical users in order to provide appropriate support [3]. When existing HIT is not reliably accessible and fully functional it becomes an impediment rather than a helpful tool [4]. The current state of computers and clinical applications used in Suite 4 at SVUH makes them unfit for efficient delivery of care; the aim of the resulting clinical audit (audit no 3548) was to clarify user needs to facilitate infrastructure support, and comply with published JCI MOI (Joint Commission International, Management of Information) and HIQA (Health Information and Quality Authority) standards [1,5].

**Methods**

Members of each clinical team using the suite were contacted regarding their HIT needs and issues encountered. After systematic review of each computer in Suite 4, the results were organized in a spreadsheet.

**Results**

Applications necessary for patient management are installed, however, numerous issues were noted to cause delays in required use of HIT such as duplicate and non-functional shortcuts (figure 1). During review, breaches of GDPR such as failures to logout of applications with access to patient information were also noted.

**Conclusions**

While it is evident that the HIT infrastructure is extremely dated and the application interfaces are not user-friendly, it is necessary to ensure the current infrastructure available is fit for purpose.

**Acknowledgement**

Ms. Claire Rutherford, consultant breast surgeon, very kindly sponsored the associated clinical audit.

**References**

1. Joint Commission International Accreditation Standards for Hospitals [Internet]. Available from: https://www.jointcommissioninternational.org/-/media/jci/jci-documents/accreditation/hospital-and-amc/jci-errata-standards-only_7th-ed-hospital.pdf
2. Sasaki N, Yamaguchi N, Okumura A, Yoshida M, Sugawara H, Imanaka Y. Does hospital information technology infrastructure promote the implementation of clinical practice guidelines? A multicentre observational study of Japanese hospitals. BMJ Open [Internet]. 2019 Jun 14 [cited 2022 Mar 2];9(6):e024700. Available from: https://www.ncbi.nlm.nih.gov/pmc/articles/PMC6588970/
3. Walsh B, Mac Domhnaill C, Mohan G. Developments in healthcare information systems in Ireland and internationally. 2021 Jun 23; Available from: https://www.esri.ie/system/files/publications/SUSTAT105_0.pdf
4. Wolfe L, Chisolm MS, Bohsali F. Clinically Excellent Use of the Electronic Health Record: Review. JMIR Human Factors. 2018 Oct 5;5(4):e10426.
5. Health Information and Quality Authority. National Standards for Safer Better Healthcare. (2012, June 26). Dublin. Available from: https://www.hiqa.ie/sites/default/files/2017-01/Safer-Better-Healthcare-Standards.pdf

**Fig. 1 (abstract A14).**
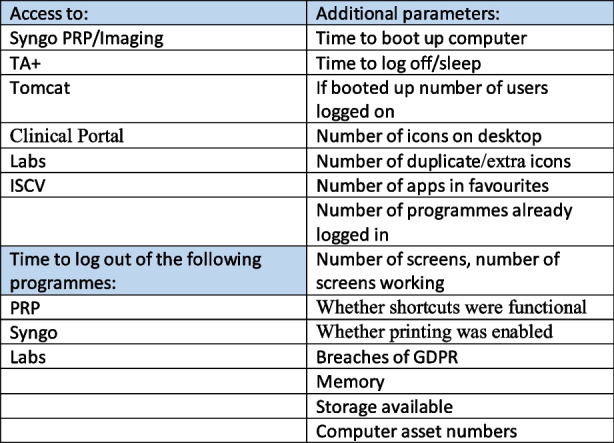
Application needs and issues reported to cause delay in clinical use of HIT

## A15. The effects of background audio-visual processing on the TMS measures of cortical excitability for biomarker research in ALS

### Yasmine S. Tadjine^1^, Friedemann Awiszus^2^, Mark Heverin^1^, Bahman Nasseroleslami^1^, Orla Hardiman^1,3^, Roisin McMackin^1^

#### ^1^ Academic Unit of Neurology, Trinity Biomedical Sciences Institute, Trinity College Dublin, University of Dublin, Ireland; ^2^ Department of Orthopedic Surgery, Otto-von-Guericke University, 44 Leipziger Strasse, 39120, Magdeburg, Germany; ^3^ Beaumont Hospital, Dublin, Ireland

*BMC Proceedings 2023,* **17(Suppl 18):**A15

**Background**

Threshold-tracking transcranial magnetic stimulation (TT-TMS) measures have been shown to be of diagnostic and prognostic value in ALS[1]. TT-TMS is increasingly being used to measure central motor pathophysiology in ALS. These studies require participants to sit still to consistently maintain very low amplitude EMG in the target muscle. However, unlike MRI studies [2], TMS study protocol typically does not allow radio or film to occupy the participant, helping them to relax and prevent potential influence of participants attending to the pulse sound or effect[3].

**Aim**

To investigate whether watching or listening to a documentary affects commonly-studied TMS measures of motor cortical excitability.

**Methods**

Data were collected from 10 healthy controls. EMG was recorded from both dominant and non-dominant hands while automated TT-TMS was used to measure resting motor threshold (RMT), threshold hunting target (THT), short and long intracortical inhibition (SICI/LICI) and interhemispheric inhibition (IHI). Stimuli were delivered to the motor cortex contralateral to the dominant hand. In the case of IHI, the conditioning stimuli were delivered to the motor cortex ipsilateral to the dominant hand. The effects of stimulation on baseline EMG amplitudes were investigated.

**Results**

RMT, SICI, LICI and IHI were not found to be significantly altered by auditory (p>0.16) or audiovisual (p>0.46) input. RMT and IHI ICC values are similar for values compared under each sensory stimulation condition.

**Conclusions**

We found no evidence of an effect of audiovisual stimulation on common TMS measures, suggesting a potential use in future.

**References**

1. Vucic S, Kiernan MC. Utility of transcranial magnetic stimulation in delineating amyotrophic lateral sclerosis pathophysiology. Handbook of clinical neurology. 2013 Jan 1;116:561-75.
2. Netzke-Doyle V. Distraction strategies used in obtaining an MRI in pediatrics: A review of the evidence. Journal of Radiology Nursing. 2010 Sep 1;29(3):87-90.
3. Avenanti A, Annella L, Candidi M, Urgesi C, Aglioti SM. Compensatory plasticity in the action observation network: virtual lesions of STS enhance anticipatory simulation of seen actions. Cerebral cortex. 2013 Mar 1;23(3):570-80.

## A16. Quantitative differences in volumetric calculations for radiation dosimetry in segmental Y90 treatment planning using hybrid angiography-CT (angio-CT) compared with anatomic segmentation

### Salma Youssef^1^, Daniel H Kwak^2^, Alex Lionberg^2^, Mikin Patel^2^, Karan Nijhawan^2^, Spencer Martens^2^, Qian Yu^2^, David Cao^3^, Osman Ahmed^2^

#### ^1^School of Medicine, University College Dublin, Belfield, Dublin, Ireland; ^2^Department of Radiology, Section of Interventional Radiology, The University of Chicago Medical Center, 5841 S. Maryland Ave, Chicago, Illinois, USA; ^3^The University of Chicago Pritzker School of Medicine, 924 E. 57^th^ St, Chicago, Illinois, USA

##### **Correspondence:** Salma Youssef (svy2004@gmail.com, salma.youssef@ucdconnect.ie)

*BMC Proceedings 2023,* **17(Suppl 18):**A16

**Purpose**

To compare treatment volumes reconstructed from hybrid Angio-CT catheter-directed infusion imaging and Couinaud anatomic model as well as the implied differences in Y-90 radiation dosimetry.

**Materials and Methods**

Patients who underwent transarterial radioembolization (TARE) using Y-90 glass microspheres with pretreatment CT or MRI imaging as well as intraprocedural angiography-CT (Angio-CT) were analyzed. Treatment volumes were delineated using both tumoral angiosomes (derived from Angio-CT) and Couinaud anatomic landmarks. Segmental and lobar treatment volumes were calculated via semi-automated contouring software. Volume and dose differences were compared by two-tailed Student’s t test or Wilcoxon signed-rank test. Factors affecting volume and dose differences were assessed via simple and/or multiple variable linear regression analysis.

**Results**

From September 2018 to March 2021, 44 patients underwent 45 lobar treatments and 38 patients received 56 segmental treatments. Tumor sizes ranged between 1.1 and 19.5-cm in diameter. Segmental volumes and treatment doses were significantly different between the Couinaud and Angio-CT volumetry methods (316 vs. 404 mL, p < 0.0001 and 253 vs. 212 Gy, p < 0.01, respectively). Watershed tumors were significantly correlated with underestimated volumes by the Couinaud anatomic model (p < 0.001). There was a significant linear relationship between tumor diameter and percent volume difference (R 2 = 0.44, p < 0.0001). The Couinaud model overestimated volumes for large tumors that exhibited central hypovascularity/necrosis and for superselected peripheral tumors.

**Conclusions**

Angio-CT may confer advantages over the Couinaud anatomic model and enable more accurate, personalized dosimetry for TARE.

## A17. Viable disc allograft supplementation in patients with chronic low back pain (VAST trial): interim 36-month results of an open-label extension study

### Salma Youssef^1^, Douglas P. Beall^2^, Jacob W Fleming^2^

#### ^1^School of Medicine, University College Dublin, Belfield, Dublin, Ireland; ^2^Clinical Radiology of Oklahoma, Oklahoma City, OK, USA

##### **Correspondence:** Salma Youssef (svy2004@gmail.com, salma.youssef@ucdconnect.ie)

*BMC Proceedings 2023,* **17(Suppl 18):**A17

**Introduction**

Degenerative disc disease (DDD) is the most common cause of chronic lower back pain (CLBP). Clinical improvements in pain and function were achieved at 12 months in both the investigational intradiscal allograft and saline groups of the VAST randomized controlled trial (NCT03709901). Here, we report outcomes in an open-label extension study at the 36-month follow-up.

**Methods**

Of 218 patients with 1 or 2-level lumbar DDD and refractory CLBP in the VAST trial, 50 continued in the 36-month extension study. In this analysis, we assessed the mean change from baseline in VAS and ODI scores and the categorical responder status. We compared this data to prior time points in this completer population only.

**Results**

Outcome data was entered for 50 patients at 36 months (allograft-treated, n=46; saline-treated, n=4). In the allograft-treated group, change from baseline in VAS (mean [95% CI]) at month 36 was -35.35 (± 25.39), with 60% of patients reporting ≥ 50% improvement in pain and >70% of patients had ≥20-point reduction in ODI in VAS at 36 months.

**Conclusion**

Patients treated with viable disc tissue allograft for degenerated lumbar discs showed sustained clinical benefits at 3 years following treatment. This interim analysis suggests viable disc tissue allograft as a durable nonsurgical treatment for patients with CLBP due to DDD.

**Acknowledgements**

Acknowledgement is made to VIVEX Biologics, Inc. (Miami, FL) for support and funding of the study.

